# Prognostic value of preoperative anorectal manometry parameters for anastomotic leakage after sphincter-preserving surgery for rectal cancer

**DOI:** 10.1007/s00384-026-05111-z

**Published:** 2026-02-25

**Authors:** Jin Gi Chang, Eon Bin Kim, Chan Wook Kim, Yong Sik Yoon, Jong Lyul Lee, In Ja Park, Seok-Byung Lim

**Affiliations:** 1 Division of Colon and Rectal Surgery, Department of Surgery, Asan Medical Center, University of Ulsan College of Medicine, 88, Olympic-Ro 43-Gil, Songpa-Gu, Seoul, 05505 Korea; 2 Department of Surgery, Dongguk University Ilsan Hospital, Dongguk University College of Medicine, Goyang, Korea

**Keywords:** Colorectal surgery, Anastomotic leak, Rectal neoplasms, Risk factors, Surgical stomas

## Abstract

**Purpose:**

Anastomotic leakage (AL) remains a serious complication following low anterior resection (LAR) for rectal cancer. Although several risk factors for AL have been identified, the role of preoperative anal sphincter function remains unexplored. We hypothesized that elevated maximum resting pressure (MRP) and maximum squeeze pressure (MSP), measured preoperatively via anorectal manometry (ARM), might increase AL risk by inducing functional outlet obstruction.

**Methods:**

This single-center retrospective cohort study included patients who underwent LAR without a diverting stoma between January 2010 and December 2015. We analyzed the associations between preoperative ARM parameters and early major AL events. Independent predictors of AL were also identified. Receiver operating characteristic curve analysis was performed to evaluate the predictive value of ARM parameters for AL.

**Results:**

Among 1,396 patients, early major AL occurred in 41 (2.9%). Patients with AL demonstrated significantly higher median MRP (55.7 vs. 42.6 mm Hg, *p* = 0.001) and MSP (186.5 vs. 150.3 mm Hg, *p* = 0.008) values. Multivariable analysis revealed that higher MRP (odds ratio [OR], 1.021 per mm Hg increase; 95% confidence interval [CI], 1.004–1.039; *p* = 0.017) and shorter tumor distance from the anal verge (OR, 0.815 per cm; 95% CI, 0.718–0.925; *p* = 0.002) were independent predictors of AL. An optimal MRP cutoff value of 55.65 mm Hg yielded 53.7% sensitivity and 75.1% specificity (area under the curve, 0.657).

**Conclusion:**

Preoperative MRP is an independent predictor of early major AL after LAR. Elevated resting anal pressure may create functional outlet obstruction, increasing intraluminal pressure at the anastomotic site and compromising healing. Preoperative ARM could identify high-risk patients who may benefit from protective interventions.

## Introduction

Rectal cancer is one of the most common gastrointestinal malignancies worldwide, with total mesorectal excision (TME) as the standard surgical approach for middle and lower rectal cancers [1, 2]. Despite advances in surgical techniques and perioperative care, anastomotic leakage (AL) remains one of the most feared complications following rectal cancer surgery, affecting both short-term morbidity and long-term outcomes [3, 4]. The incidence of AL after low anterior resection (LAR) has been reported to range from 4 to 20%, depending on the definitions applied and the method of detection [5–8]. Regardless of severity, AL is significantly associated with increased mortality, reoperation rates, and hospital costs [8]. Furthermore, it impairs long-term anorectal function and may compromise oncologic survival [9].

Despite extensive research on risk factors for AL, the role of anal sphincter function—as measured via anorectal manometry (ARM)—remains largely unexplored. ARM objectively quantifies sphincter pressures [10]. The internal anal sphincter maintains the maximum resting pressure (MRP), preventing passive fecal spillage, while the external anal sphincter generates the maximum squeeze pressure (MSP) for voluntary control. Although ARM is well established for predicting postoperative functional outcomes such as fecal incontinence or LAR syndrome, its potential role in predicting AL has not yet been investigated [11].

We hypothesized that elevated anal sphincter pressure might increase intraluminal pressure at the anastomotic site, potentially compromising staple line integrity. Therefore, the present study aimed to evaluate whether preoperative ARM parameters are associated with AL after LAR.

## Methods

### Study design & patient selection

This single-center retrospective cohort study was conducted at Asan Medical Center. We screened consecutive patients who underwent TME with LAR for rectal cancer between January 2010 and December 2015. Preoperative workup included colonoscopy, abdominopelvic/chest CT, and rectal MRI. Liver MRI or PET-CT was performed to evaluate suspected metastases. Patients were included if they underwent elective LAR with primary double-stapling anastomosis below the peritoneal reflection. Exclusion criteria included palliative surgery, prior neoadjuvant chemoradiotherapy, TME without primary anastomosis (e.g., Hartmann’s procedure or abdominoperineal resection), construction of a diverting stoma, and incomplete preoperative anorectal manometry data. Additionally, patients who underwent intersphincteric resection were excluded to minimize confounding related to postoperative alterations in anal sphincter function.

### Data collection and definitions

Clinical and pathological data were extracted from the patients’ electronic medical records. The collected clinicopathological variables included age, sex, diabetes mellitus, hypertension, body mass index (BMI), tumor distance from the anal verge, type of operation, surgical approach (open vs. minimally invasive surgery), T stage, N stage, and postoperative early major AL events. Early major AL was defined according to the International Study Group of Rectal Cancer (ISREC) grading system as grade C leakage occurring within 30 days after surgery [3]. Preoperative ARM parameters were obtained from manometry reports and included high-pressure zone length (HPZL), MRP, and MSP.

### Surgical procedures

During the study period, all surgical procedures were performed by eight board-certified colorectal surgeons using open, laparoscopic, or robotic approaches. Patients underwent mechanical bowel preparation, with prophylactic antibiotics administered preoperatively and discontinued within 24 h postoperatively. LAR involved high or low ligation of the inferior mesenteric artery and total or tumor-specific mesorectal excision, followed by double-stapled anastomosis. The extent of mesorectal excision was determined based on tumor location; TME was performed for mid-to-low rectal cancers, whereas tumor-specific mesorectal excision was applied for upper rectal and rectosigmoid cancers. The creation of a protective diverting stoma was determined intraoperatively at the surgeon’s discretion, based on patient-related risk factors and intraoperative findings. Transanal decompression tubes were not used in this study cohort.

### Anorectal manometry

Preoperative ARM was routinely performed for candidates of sphincter-preserving surgery, typically within one month before the surgery. The test was performed using a Microcapillary Infusion System (J.S. Biomedicals Inc., USA) via the distilled water infusion method. A 5-mm-diameter catheter equipped with eight radially oriented pressure channels—positioned 5 cm from the catheter tip—was used, with distilled water perfused at a constant rate of 0.5 mL/channel/min. Data were recorded and analyzed using LGI Polygram software (Synetics Liberty System, USA) to calculate key parameters, including vector volume, sphincter pressures, and HPZL. Patients were placed in the left lateral decubitus position. Following zero-point calibration at the anal verge, the catheter was inserted such that the pressure channels were positioned 6 cm above the anal verge. Resting and squeeze pressures were alternately measured in triplicate using a rapid pull-through technique at a constant speed of 1 cm/s. MRP and MSP were calculated, with the latter defined as the mean of the peak pressures recorded during voluntary squeeze maneuvers. Subsequently, stationary pull-through testing was conducted at 1-cm intervals. Rectal sensory parameters, including minimal sensory volume and maximal tolerance volume, was assessed using a balloon catheter inflated incrementally by 60 mL. The reference normal ranges were as follows: HPZL, 2.0–3.5 cm; MRP, 40–70 mm Hg; and MSP, 100–180 mm Hg.

### Study outcomes

The primary outcome was the association between preoperative ARM parameters (i.e., HPZL, MRP, and MSP) and the incidence of early major AL. Secondary outcomes included identifying independent risk factors (from clinicopathological and preoperative ARM parameters) for AL and assessing the predictive performance of preoperative ARM parameters.

### Statistical analysis

Continuous variables were presented as mean ± standard deviation (SD) or median with interquartile range (IQR), as appropriate. Categorical variables were expressed as frequencies and percentages. Between-group comparisons were performed using Student’s *t* test or the Mann–Whitney U test for continuous data, and Pearson’s chi-square test or Fisher’s exact test for categorical data. Logistic regression analyses were conducted to identify independent risk factors for AL. Variables with clinical significance, including key manometric parameters, were entered into the multivariable model. The predictive performance of preoperative ARM parameter was evaluated using receiver operating characteristic (ROC) curve analysis. The area under the ROC curve (AUC) and its 95% confidence interval (CI) were calculated. The optimal cutoff value was determined using the Youden index, and the corresponding sensitivity and specificity were reported. A two-sided p value < 0.05 was considered statistically significant. All statistical analyses were performed using R software (version 4.5.1; R Foundation for Statistical Computing, Vienna, Austria).

## Results

### Clinicopathological characteristics

**Fig. 1:**
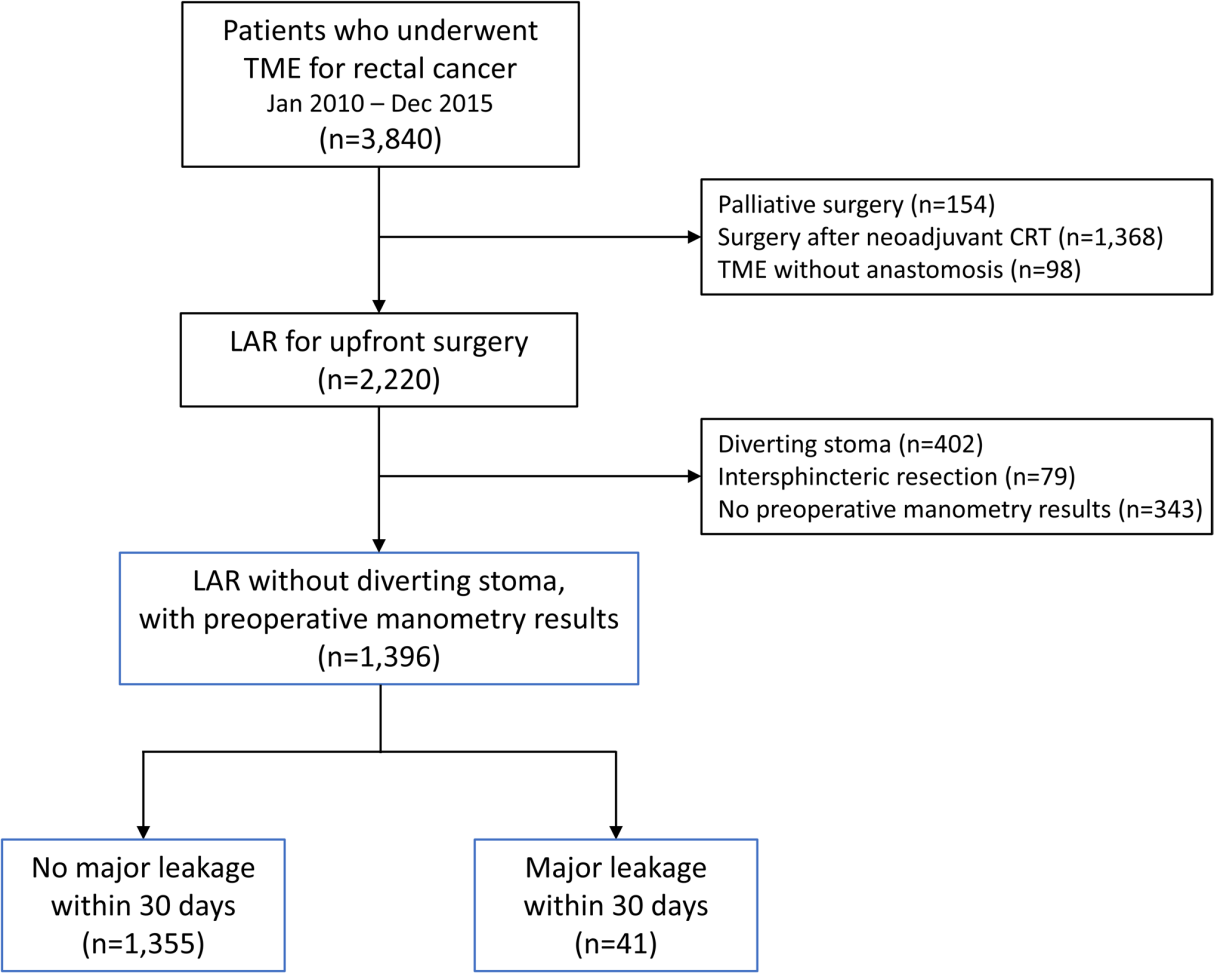
Patient selection flow diagram; TME, total mesorectal excision; CRT, chemoradiotherapy; LAR, low anterior resection

Of the 3,840 patients who underwent LAR for rectal cancer, 1,396 were enrolled (Fig. 1). Among this cohort, early major AL occurred in 41 patients (2.9%). The median time to AL diagnosis was 6 days (IQR, 3–13 days). Table 1 summarizes baseline clinicopathological characteristics. Patients who developed AL were younger (57.1 ± 11.7 vs. 60.9 ± 10.5 years, *p* = 0.024) and had a significantly shorter tumor distance from the anal verge (7.7 ± 2.4 vs. 9.1 ± 2.7 cm, *p* = 0.002), compared with patients without AL. There were no significant differences in sex, comorbidities, or tumor stage between the groups.

**Table 1:** Clinicopathological characteristics

Variables	Total (*n* = 1,396)	Non-AL (*n* = 1,355)	AL (*n* = 41)	*p*
Sex				0.369
Female, no. (%)	554 (39.7)	541 (97.7)	13 (2.3)	
Male, no. (%)	842 (60.3)	814 (96.7)	28 (3.3)	
Age (years), mean (SD)	60.8 (10.5)	60.9 (10.5)	57.1 (11.7)	0.024
Diabetes mellitus, no. (%)	183 (13.1)	180 (98.4)	3 (1.6)	0.379
Hypertension, no. (%)	485 (34.7)	471 (97.1)	14 (2.9)	1.000
BMI (kg/m2), mean (SD)	23.8 (3.0)	23.8 (3.0)	23.0 (3.2)	0.111
Tumor distance from AV (cm), mean (SD)	9.0 (2.7)	9.1 (2.7)	7.7 (2.4)	0.002
Approach type				0.794
Open, no. (%)	704 (50.4)	682 (96.9)	22 (3.1)	
MIS, no. (%)	692 (49.6)	673 (97.3)	19 (2.7)	
T stage				0.647
T0-T2, no. (%)	507 (36.3)	494 (97.4)	13 (2.6)	
T3-T4, no. (%)	889 (63.7)	861 (96.9)	28 (3.1)	
N stage				1.000
N0, no. (%)	828 (59.3)	804 (97.1)	24 (2.9)	
N +, no. (%)	568 (40.7)	551 (97.0)	17 (3.0)	

*AL* anastomotic leakage, *AV* anal verge, *BMI* body mass index, *MIS* minimally invasive surgery, *SD* standard deviation

### Preoperative ARM parameters

**Fig. 2:**
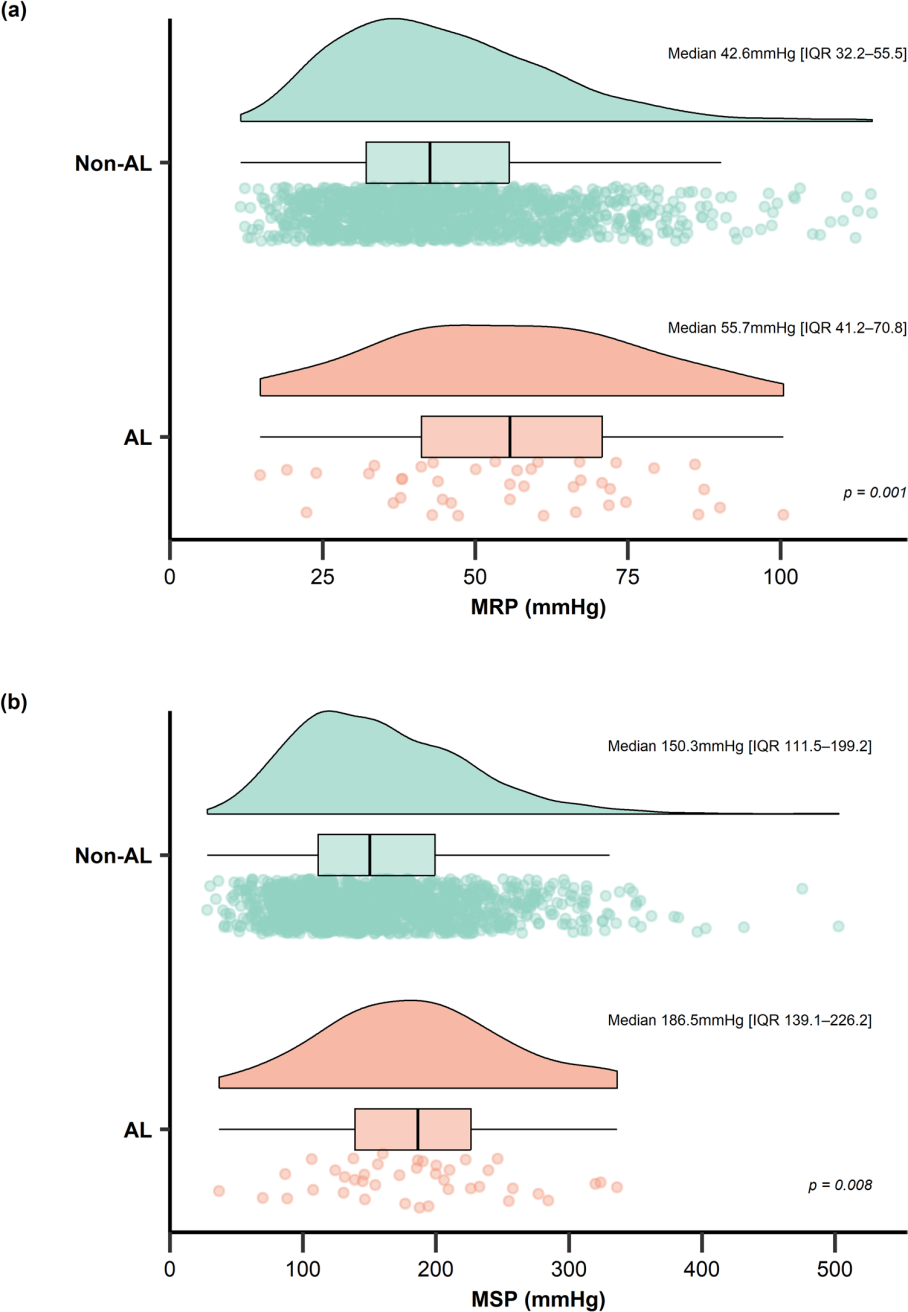
Raincloud and box plots of preoperative anorectal manometric parameters by early anastomotic leakage (AL) status. **a** Maximum resting pressure (MRP). **b** Maximum squeeze pressure (MSP); IQR, interquartile range

When preoperative ARM parameters were compared by AL status, median HPZL did not differ significantly between the groups (Table 2); however, both median MRP and MSP were significantly higher in the patients with AL (*p* = 0.001 and 0.008, respectively). As illustrated in Fig. 2, both MRP and MSP showed right-shifted distributions in the AL group compared with the non-AL group.

**Table 2:** Comparison of preoperative anorectal manometry parameters by anastomotic leakage status

Parameters	Total (*n* = 1,396)	Non-AL (*n* = 1,355)	AL (*n* = 41)	*p*
HPZL (cm)	2.2 (1.8–2.7)	2.2 (1.8–2.7)	2.3 (2.0–2.6)	0.461
MRP (mm Hg)	42.8 (32.3–56.2)	42.6 (32.2–55.5)	55.7 (41.2–70.8)	0.001
MSP (mm Hg)	151.1 (111.9–200.2)	150.3 (111.5–199.2)	186.5 (139.1–226.2)	0.008

*AL* anastomotic leakage, *HPZL* high-pressure zone length, *MRP* maximum resting pressure, *MSP* maximum squeeze pressure

*Data are presented as median (interquartile range)

### Risk factors for early AL

Univariable logistic regression analysis revealed that age, preoperative MRP, preoperative MSP, and tumor distance from the anal verge were significantly associated with AL (Table 3). In multivariable analysis with these variables, higher preoperative MRP (odds ratio [OR], 1.021; 95% CI, 1.004–1.039; *p* = 0.017) and shorter tumor distance from the anal verge (OR, 0.815; 95% CI, 0.718–0.925; *p* = 0.002) were identified as independent risk factors for AL.

**Table 3:** Univariable and multivariable logistic regression analyses for early anastomotic leakage

	Univariable			Multivariable		
Variables	OR	95% CI	*p*	OR	95% CI	*p*
Age	0.967	0.939–0.996	0.025	0.989	0.956–1.022	0.505
Male (vs. Female)	1.431	0.735–2.788	0.292			
Diabetes mellitus	0.515	0.157–1.687	0.273			
Hypertension	0.973	0.505–1.874	0.935			
BMI	0.915	0.820–1.020	0.110			
MRP	1.028	1.013–1.044	<0.001	1.021	1.004–1.039	0.017
MSP	1.006	1.001–1.01	0.010	1.003	0.999–1.008	0.158
T3-T4 (vs. T0-T2)	1.236	0.634–2.408	0.534			
N + (vs. N0)	1.034	0.550–1.942	0.918			
Tumor distance from AV	0.816	0.719–0.926	0.002	0.815	0.718–0.925	0.002
MIS (vs. open surgery)	0.875	0.469–1.632	0.675			

*AV* anal verge, *BMI* body mass index, *CI* confidence interval, *MIS* minimally invasive surgery, *MRP* maximum resting pressure, *MSP* maximum squeeze pressure, *OR* odds ratio

### Predictive performance of preoperative MRP and MSP

**Fig. 3:**
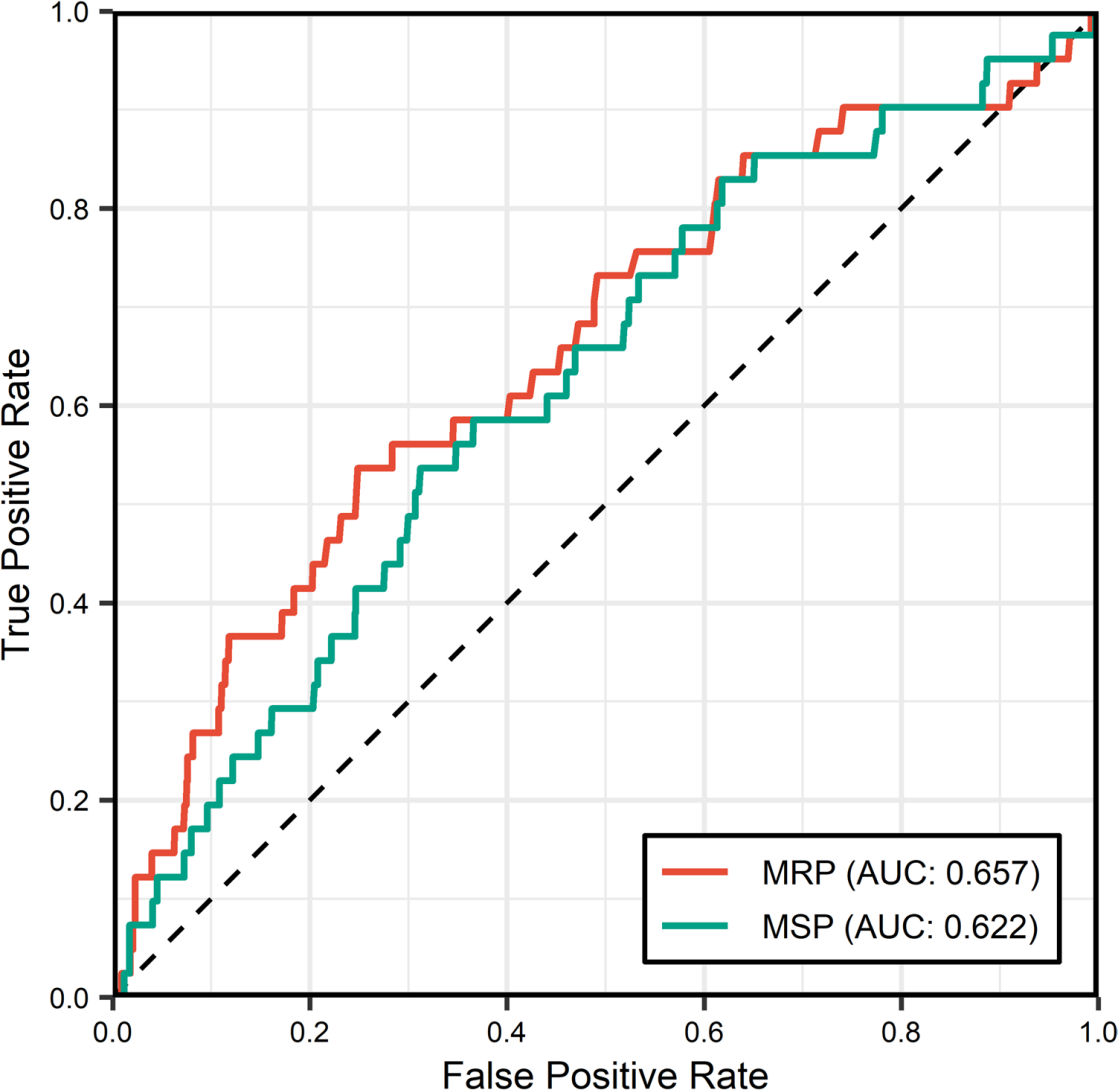
Receiver operating characteristic curve of preoperative maximum resting pressure (MRP) and maximum squeeze pressure (MSP) for predicting anastomotic leakage; AUC, area under the curve

ROC curve analysis was performed to evaluate the predictive performance of preoperative MRP and MSP for early major AL (Fig. 3). For MRP, the AUC was 0.657 (95% CI, 0.565–0.749), with an optimal cutoff value of 55.65 mm Hg yielding a sensitivity of 53.7% and a specificity of 75.1% for predicting major AL. For MSP, the AUC was 0.622 (95% CI, 0.538–0.710), with an optimal cutoff value of 185.45 mm Hg yielding a sensitivity of 53.7% and a specificity of 68.8% for predicting major AL.

### Clinicopathological characteristics stratified by MRP

Patients were categorized into low (< 55.65 mmHg) and high (≥ 55.65 mmHg) MRP groups based on the cutoff value derived from ROC curve analysis (Table 4). The high MRP group was significantly younger (54.9 vs. 62.8 years, *p* < 0.001) and had a higher mean BMI (24.2 vs. 23.6 kg/m^2^, p = 0.002) compared to the low MRP group. The prevalence of comorbidities, including diabetes mellitus and hypertension, was significantly lower in the high MRP group. Tumor-related characteristics, including distance from the anal verge (9.2 vs. 9.0 cm, p = 0.267) and tumor stage, did not differ significantly between the two groups. The high MRP group exhibited a significantly higher incidence of early AL (6.1% vs. 1.8%, p < 0.001).

**Table 4:** Clinicopathological characteristics stratified by the MRP cutoff value of 55.65 mmHg

Variables	Low MRP group (*n* = 1,037)	High MRP group (*n* = 359)	*p*
Sex			0.262
Female, no. (%)	421 (40.6)	133 (37.0)	
Male, no. (%)	616 (59.4)	226 (63.0)	
Age (years), mean (SD)	62.8 (9.9)	54.9 (10.0)	< 0.001
Diabetes mellitus, no. (%)	161 (15.5)	22 (6.1)	< 0.001
Hypertension, no. (%)	386 (37.2)	99 (27.6)	0.001
BMI (kg/m2), mean (SD)	23.6 (2.9)	24.2 (3.2)	0.002
Tumor distance from AV (cm), mean (SD)	9.0 (2.6)	9.2 (2.8)	0.267
Approach type			< 0.001
Open, no. (%)	555 (53.5)	149 (41.5)	
MIS, no. (%)	482 (46.5)	210 (58.5)	
T stage			0.787
T0-T2, no. (%)	374 (36.1)	133 (37.0)	
T3-T4, no. (%)	663 (63.9)	226 (63.0)	
N stage			0.762
N0, no. (%)	618 (59.6)	210 (58.5)	
N +, no. (%)	419 (40.4)	149 (41.5)	
Early AL, no. (%)	19 (1.8)	22 (6.1)	< 0.001

*AL* anastomotic leakage, *AV* anal verge, *BMI* body mass index, *MIS* minimally invasive surgery, *MRP* maximum resting pressure, *SD* standard deviation

## Discussion

This retrospective cohort study demonstrated that preoperative MRP measured via ARM was an independent predictor of early major AL. This finding supports our hypothesis that elevated anal resting pressure may increase the risk of AL through a mechanism of functional outlet obstruction. During the early postoperative period, when anastomotic healing remains incomplete, high anal sphincter tone may impede the passage of stool and gas, leading to increased intraluminal pressure proximal to the anastomosis and elevated mechanical stress on the suture line. This proposed mechanism is consistent with evidence from other gastrointestinal surgical settings, where outlet obstruction or elevated intraluminal pressure has been implicated in anastomotic failure. For example, in esophageal surgery, delayed gastric emptying and pyloric dysfunction increase intragastric pressure and are associated with higher rates of esophagogastric AL [12]. In addition, previous studies have shown that transanal tube placement reduces the incidence of AL by decreasing intraluminal pressure at the anastomotic site [13–15]. Collectively, these observations reinforce our proposed pathophysiological model, wherein elevated preoperative anal resting pressure predisposes patients to AL through functional outlet obstruction, particularly during the critical early postoperative phase of anastomotic healing.

To our knowledge, this is the first study to investigate the pathophysiological impact of preoperative ARM parameters on anastomotic integrity following rectal cancer surgery. Previous applications of manometry in colorectal surgery have focused primarily on predicting postoperative functional outcomes—particularly fecal incontinence and LAR syndrome—rather than surgical complications [11, 16, 17]. Several studies have demonstrated that lower preoperative MRP or MSP values are associated with worse postoperative continence after intersphincteric resection or very LAR [17, 18]. However, the potential role of sphincter pressure in the mechanism of anastomotic failure has not been previously explored. Our finding that elevated preoperative MRP is significantly associated with AL represents a conceptually distinct clinical implication. While low sphincter pressures indicate functional weakness that may compromise continence, increased resting pressure may act as a functional outlet obstruction, leading to elevated proximal intraluminal pressure. This pressure gradient is particularly relevant during the immediate postoperative period, when the anastomosis has minimal tensile strength and is therefore highly vulnerable to mechanical disruption [19].

Various factors contribute to the risk of AL. [20]. While male sex, high BMI, and comorbidities are well-known predictors, tumor height remains one of the most robust factors [21–23]. A low anastomosis is associated with a significantly increased risk of AL, largely owing to greater technical complexity and compromised anastomotic perfusion [24–26]. Our findings are concordant with these previous reports. In this study, a shorter tumor distance from the anal verge was associated with a higher incidence of AL, demonstrating a pattern consistent with prior literature indicating increased risk of anastomotic complications in more distal tumors. In multivariable analysis, shorter tumor distance from the anal verge remained an independent risk factor after adjustment for established covariates. This result further reinforces the well-recognized association between shorter tumor distance from the anal verge and an increased risk of AL.

Contrary to general expectations, traditional risk factors such as male sex, BMI, and diabetes mellitus were not identified as independent predictors of AL in our series. This finding likely resulted from the exclusion of two potent confounders: neoadjuvant chemoradiotherapy and diverting stoma formation. Neoadjuvant chemoradiotherapy, in particular, has been reported to potentially alter sphincter tone [27, 28] and, although controversial, may independently influence AL risk [29, 30]. By excluding patients who received neoadjuvant treatment, we aimed to isolate the independent effect of baseline sphincter pressure on anastomotic outcomes. The exclusion of these factors also yielded a lower overall leakage rate (2.9%) compared with that reported in unselected clinical series (4–13%) [6, 7]. While this limits direct generalizability to routine clinical practice, it represents a methodological strength by minimizing confounding and allowing a more precise assessment of the independent contribution of anal sphincter pressure to AL risk. In this context, it is important to recognize that anal sphincter pressures vary by patient demographics; thus, the effects of sex and age on AL risk may be partially mediated through baseline pressure. Previous studies have shown that MRP is higher in males and declines with advancing age [31, 32]. These physiological patterns may suggest a new explanation for the consistently higher incidence of AL observed in male patients. Similarly, the age-dependent decline in sphincter pressure may help explain why patients who developed AL in this study were younger than those without AL. These demographic patterns are further supported by the comparison of characteristics stratified by the MRP cutoff. The high MRP group was significantly younger and had fewer comorbidities, consistent with the known negative correlation between age and sphincter pressure. Notably, although the tumor distance from the anal verge was comparable between the two groups, the incidence of AL was significantly higher in patients with high MRP (6.1% vs. 1.8%). This finding suggests that elevated anal tone appears to be a distinct risk factor, exerting a mechanical impact on the anastomosis independent of tumor location.

Although elevated MSP was hypothesized as a potential risk factor and showed a significant association with AL in univariable analysis (p = 0.010), it failed to demonstrate independent significance in the multivariable model (p = 0.158). This distinction is likely due to the physiological difference between the two ARM parameters. While high MRP may exert continuous intraluminal pressure and sustain physical stress on the anastomotic line, MSP reflects only voluntary, intermittent contractions, which may have a limited impact on AL [33, 34]. Furthermore, the correlation between MRP and MSP may have influenced this result. Prior studies reported that both MRP and MSP were significantly higher in male and younger patients [31, 32]. In addition, patients with sarcopenia have shown concomitant reductions in both MRP and MSP [35]. Accordingly, the higher MSP observed among patients who developed AL may represent a secondary effect driven by its correlation with MRP, rather than indicating that MSP itself is an independent predictor of AL.

The findings of this study have important clinical implications for surgical decision-making in rectal cancer. Preoperative ARM can serve as a supplementary screening tool for preoperative risk stratification. Surgeons may incorporate high preoperative MRP as a complementary tool when determining individualized AL-preventing strategies for each patient. Construction of a diverting stoma can be selectively considered to minimize the consequences of potential AL. When stoma creation is not feasible or is declined by the patient, alternative protective strategies are recommended—for example, transanal drainage tube placement to reduce intraluminal pressure [13–15] or staple line reinforcement techniques, such as circumferential oversewing, to mechanically strengthen the anastomosis [36–38].

This study has several limitations. First, selection bias exists owing to the exclusion of patients with diverting stomas and those receiving neoadjuvant chemoradiotherapy. This limits the generalizability of our findings to high-risk or irradiated populations and may lead to an underestimation of the true incidence of AL. Additionally, the exclusive focus on ISREC Grade C leakage precludes the assessment of risk factors associated with Grade A or B leakage. Second, the proposed mechanism of functional outlet obstruction remains inferential, as we did not directly measure postoperative intraluminal pressures. Third, other relevant surgical factors, such as the number of linear stapler firings, anastomotic level, tension, and objective assessments of anastomotic perfusion were not analyzed. Moreover, the potential influence of mechanical anal dilatation before transanal stapler insertion on sphincter function was not evaluated. Finally, the modest AUC indicates that MRP has limited discriminative ability as a standalone predictor and should therefore be interpreted within the context of a multivariable risk assessment. Future research should focus on external validation in prospective, multicenter cohorts to confirm generalizability. In addition, mechanistic studies incorporating postoperative intraluminal pressure measurements could help clarify whether functional outlet obstruction contributes to AL risk. Furthermore, integrating MRP into comprehensive risk prediction models may improve clinical utility and inform the use of selective interventions, such as diverting stomas or alternative techniques.

## Conclusion

Our findings demonstrated that elevated preoperative MRP is independently associated with early major AL after sphincter-preserving surgery for rectal cancer. Preoperative ARM may therefore offer clinically meaningful prognostic value and, following further validation, could assist in identifying patients who are most likely to benefit from targeted protective interventions.

## Data Availability

The data that support the findings of this study are available from the corresponding author upon reasonable request.

## References

[1] Siegel RL, Miller KD, Wagle NS, Jemal A (2023) Cancer statistics, 2023. CA Cancer J Clin 73:17–48. 10.3322/caac.21763 36633525 10.3322/caac.21763

[2] Heald RJ, Husband EM, Ryall RD (1982) The mesorectum in rectal cancer surgery–the clue to pelvic recurrence? Br J Surg 69:613–616. 10.1002/bjs.1800691019 6751457 10.1002/bjs.1800691019

[3] Rahbari NN, Weitz J, Hohenberger W et al (2010) Definition and grading of anastomotic leakage following anterior resection of the rectum: a proposal by the International Study Group of Rectal Cancer. Surgery 147:339–351. 10.1016/j.surg.2009.10.012 20004450 10.1016/j.surg.2009.10.012

[4] Kang CY, Halabi WJ, Chaudhry OO et al (2013) Risk factors for anastomotic leakage after anterior resection for rectal cancer. JAMA Surg 148:65–71. 10.1001/2013.jamasurg.2 22986932 10.1001/2013.jamasurg.2

[5] Kawada K, Hasegawa S, Hida K et al (2014) Risk factors for anastomotic leakage after laparoscopic low anterior resection with DST anastomosis. Surg Endosc 28:2988–2995. 10.1007/s00464-014-3564-0 24853855 10.1007/s00464-014-3564-0 PMC4186976

[6] Fukada M, Matsuhashi N, Takahashi T et al (2019) Risk and early predictive factors of anastomotic leakage in laparoscopic low anterior resection for rectal cancer. World J Surg Oncol 17:178. 10.1186/s12957-019-1716-3 31677643 10.1186/s12957-019-1716-3 PMC6825709

[7] Nagaoka T, Fukunaga Y, Mukai T et al (2021) Risk factors for anastomotic leakage after laparoscopic low anterior resection: a single-center retrospective study. Asian J Endosc Surg 14:478–488. 10.1111/ases.12900 33205524 10.1111/ases.12900

[8] Kinugasa T, Nagasu S, Murotani K et al (2020) Analysis of risk factors for anastomotic leakage after lower rectal cancer resection, including drain type: a retrospective single-center study. BMC Gastroenterol 20:315. 10.1186/s12876-020-01462-1 32977772 10.1186/s12876-020-01462-1 PMC7519527

[9] Suzuki N, Yoshida S, Tomochika S et al (2021) Determining the protective characteristics and risk factors for the development of anastomotic leakage after low anterior resection for rectal cancer. Surg Today 51:713–720. 10.1007/s00595-020-02133-0 33006668 10.1007/s00595-020-02133-0 PMC8055621

[10] Rao SSC (2010) Advances in diagnostic assessment of fecal incontinence and dyssynergic defecation. Clin Gastroenterol Hepatol 8:910–919. 10.1016/j.cgh.2010.06.004 20601142 10.1016/j.cgh.2010.06.004 PMC2964406

[11] Pilkington SA, Bhome R, Gilbert S et al (2021) Sequential assessment of bowel function and anorectal physiology after anterior resection for cancer: a prospective cohort study. Colorectal Dis 23:2436–2446. 10.1111/codi.15754 34032359 10.1111/codi.15754

[12] Urschel JD, Blewett CJ, Bennett WF et al (2001) Handsewn or stapled esophagogastric anastomoses after esophagectomy for cancer: meta-analysis of randomized controlled trials. Dis Esophagus 14:212–217. 10.1046/j.1442-2050.2001.00187.x 11869322 10.1046/j.1442-2050.2001.00187.x

[13] Xiao L, Zhang W, Jiang P et al (2011) Can transanal tube placement after anterior resection for rectal carcinoma reduce anastomotic leakage rate? A single-institution prospective randomized study. World J Surg 35:1367–1377. 10.1007/s00268-011-1053-3 21437746 10.1007/s00268-011-1053-3

[14] Yang CS, Choi GS, Park JS et al (2016) Rectal tube drainage reduces major anastomotic leakage after minimally invasive rectal cancer surgery. Colorectal Dis 18:O445–O452. 10.1111/codi.13506 27611180 10.1111/codi.13506

[15] Tamura K, Matsuda K, Horiuchi T et al (2021) Laparoscopic anterior resection with or without transanal tube for rectal cancer patients – a multicenter randomized controlled trial. Am J Surg 222:606–612. 10.1016/j.amjsurg.2020.12.054 33413874 10.1016/j.amjsurg.2020.12.054

[16] Ihnát P, Slívová I, Tulinsky L et al (2018) Anorectal dysfunction after laparoscopic low anterior rectal resection for rectal cancer with and without radiotherapy (manometry study). J Surg Oncol 117:710–716. 10.1002/jso.24885 29094352 10.1002/jso.24885

[17] Kitaguchi D, Nishizawa Y, Sasaki T et al (2019) Clinical benefit of high resolution anorectal manometry for the evaluation of anal function after intersphincteric resection. Colorectal Dis 21:335–341. 10.1111/codi.14528 30537066 10.1111/codi.14528

[18] Kochi M, Egi H, Adachi T et al (2020) Preoperative incremental maximum squeeze pressure as a predictor of fecal incontinence after very low anterior resection for low rectal cancer. Surg Today 50:516–524. 10.1007/s00595-019-01926-2 31797125 10.1007/s00595-019-01926-2

[19] Thornton FJ, Barbul A (1997) Healing in the gastrointestinal tract. Surg Clin North Am 77:549–573. 10.1016/s0039-6109(05)70568-5 9194880 10.1016/s0039-6109(05)70568-5

[20] McDermott FD, Heeney A, Kelly ME et al (2015) Systematic review of preoperative, intraoperative and postoperative risk factors for colorectal anastomotic leaks. Br J Surg 102:462–479. 10.1002/bjs.9697 25703524 10.1002/bjs.9697

[21] Park JS, Choi G-S, Kim SH et al (2013) Multicenter analysis of risk factors for anastomotic leakage after laparoscopic rectal cancer excision: the Korean laparoscopic colorectal surgery study group. Ann Surg 257:665–671. 10.1097/SLA.0b013e31827b8ed9 23333881 10.1097/SLA.0b013e31827b8ed9

[22] Bertelsen CA, Andreasen AH, Jørgensen T et al (2010) Anastomotic leakage after curative anterior resection for rectal cancer: short and long-term outcome. Colorectal Dis 12:e76-81. 10.1111/j.1463-1318.2009.01935.x 19438879 10.1111/j.1463-1318.2009.01935.x

[23] Eriksen MT, Wibe A, Norstein J et al (2005) Anastomotic leakage following routine mesorectal excision for rectal cancer in a national cohort of patients. Colorectal Dis 7:51–57. 10.1111/j.1463-1318.2004.00700.x 15606585 10.1111/j.1463-1318.2004.00700.x

[24] Degiuli M, Elmore U, De Luca R et al (2022) Risk factors for anastomotic leakage after anterior resection for rectal cancer (RALAR study): a nationwide retrospective study of the Italian Society of Surgical Oncology Colorectal Cancer Network Collaborative Group. Colorectal Dis 24:264–276. 10.1111/codi.15997 34816571 10.1111/codi.15997 PMC9300066

[25] Quan Z, Lin L, Huang R et al (2025) Building a risk prediction model for anastomotic leakage postoperative low rectal cancer based on Lasso-Logistic regression. BMC Gastroenterol 25:540. 10.1186/s12876-025-04128-y 40731389 10.1186/s12876-025-04128-y PMC12309234

[26] Chen J, Zhang Z, Chang W et al (2021) Short-term and long-term outcomes in mid and low rectal cancer with robotic surgery. Front Oncol 11:603073. 10.3389/fonc.2021.603073 33767981 10.3389/fonc.2021.603073 PMC7985529

[27] Canda AE, Terzi C, Gorken IB et al (2010) Effects of preoperative chemoradiotherapy on anal sphincter functions and quality of life in rectal cancer patients. Int J Colorectal Dis 25(2):197–204. 10.1007/s00384-009-0807-y 19784660 10.1007/s00384-009-0807-y

[28] Ammann K (2003) Impact of neoadjuvant chemoradiation on anal sphincter function in patients with carcinoma of the midrectum and low rectum. Arch Surg 138:257. 10.1001/archsurg.138.3.257 12611569 10.1001/archsurg.138.3.257

[29] Yang J, Luo Y, Tian T et al (2022) Effects of neoadjuvant radiotherapy on postoperative complications in rectal cancer: a meta-analysis. J Oncol 2022:8197701. 10.1155/2022/8197701 35035483 10.1155/2022/8197701 PMC8754670

[30] Hu MH, Huang RK, Zhao RS et al (2017) Does neoadjuvant therapy increase the incidence of anastomotic leakage after anterior resection for mid and low rectal cancer? A systematic review and meta-analysis. Colorectal Dis 19:16–26. 10.1111/codi.13424 27321374 10.1111/codi.13424

[31] Lee HJ, Jung KW, Han S et al (2014) Normal values for high-resolution anorectal manometry/topography in a healthy Korean population and the effects of gender and body mass index. Neurogastroenterol Motil 26:529–537. 10.1111/nmo.12297 24387705 10.1111/nmo.12297

[32] Sharma M, Lowry AC, Rao SS et al (2021) A multicenter study of anorectal pressures and rectal sensation measured with portable manometry in healthy women and men. Neurogastroenterol Motil 33:e14067. 10.1111/nmo.14067 33462889 10.1111/nmo.14067 PMC8169521

[33] Diamant NE, Kamm MA, Wald A, Whitehead WE (1999) AGA technical review on anorectal testing techniques. Gastroenterology 116:735–760. 10.1016/s0016-5085(99)70195-2 10029632 10.1016/s0016-5085(99)70195-2

[34] Lestar B, Penninckx F, Kerremans R (1989) The composition of anal basal pressure. An in vivo and in vitro study in man. Int J Colorectal Dis 4:118–122. 10.1007/BF01646870 2746132 10.1007/BF01646870

[35] Neshatian L, Liu A, Gurland B et al (2025) Sarcopenia is an independent risk factor for the decline in anal sphincter function and increased levator laxity in women. Am J Gastroenterol. 10.14309/ajg.0000000000003544 40377232 10.14309/ajg.0000000000003544

[36] Jung J-M, Yang S, Yoon YS et al (2025) Does robotic circumferential oversewing reduce anastomotic leakage in stapled anastomosis for rectal cancer surgery? Tech Coloproctol 29:160. 10.1007/s10151-025-03207-2 40813560 10.1007/s10151-025-03207-2 PMC12354487

[37] Řezáč T, Špička P, Zbořil P et al (2022) Effect of reinforcement suture on anastomotic healing in rectal and sigmoid tumor resections, single-center experience: a retrospective case-controlled study. Ann Coloproctology 39:139–146. 10.3393/ac.2021.00948.0135 10.3393/ac.2021.00948.0135 PMC10169550 35272450

[38] Zhuang Z-X, Zhang Y, Yang X-Y et al (2025) Efficacy of reinforcing sutures in preventing anastomotic leakage after surgery for rectal cancer: a systematic review and metaanalysis. World J Gastrointest Surg 17:103758. 10.4240/wjgs.v17.i5.103758 40502517 10.4240/wjgs.v17.i5.103758 PMC12149912

